# Long-lived plasma cells are early and constantly generated in New Zealand Black/New Zealand White F1 mice and their therapeutic depletion requires a combined targeting of autoreactive plasma cells and their precursors

**DOI:** 10.1186/s13075-015-0551-3

**Published:** 2015-03-02

**Authors:** Adriano Taddeo, Laleh Khodadadi, Caroline Voigt, Imtiaz M Mumtaz, Qingyu Cheng, Katrin Moser, Tobias Alexander, Rudolf A Manz, Andreas Radbruch, Falk Hiepe, Bimba F Hoyer

**Affiliations:** Department of Rheumatology and Clinical Immunology, Charité University Hospital Berlin, Charitéplatz 1, 10117 Berlin, Germany; German Rheumatism Research Centre, a Leibniz Institute, Charitéplatz 1, 10117 Berlin, Germany; Institute for Systemic Inflammation Research, University of Luebeck, Ratzeburger Allee 160, 23562 Luebeck, Germany

## Abstract

**Introduction:**

Autoantibodies contribute significantly to the pathogenesis of systemic lupus erythematosus (SLE). Unfortunately, the long-lived plasma cells (LLPCs) secreting such autoantibodies are refractory to conventional immunosuppressive treatments. Although generated long before the disease becomes clinically apparent, it remains rather unclear whether LLPC generation continues in the established disease. Here, we analyzed the generation of LLPCs, including autoreactive LLPCs, in SLE-prone New Zealand Black/New Zealand White F1 (NZB/W F1) mice over their lifetime, and their regeneration after depletion.

**Methods:**

Bromodeoxyuridine pulse-chase experiments in mice of different ages were performed in order to analyze the generation of LLPCs during the development of SLE. LLPCs were enumerated by flow cytometry and autoreactive anti-double-stranded DNA (anti-dsDNA) plasma cells by enzyme-linked immunospot (ELISPOT). For analyzing the regeneration of LLPCs after depletion, mice were treated with bortezomib alone or in combination with cyclophosphamide and plasma cells were enumerated 12 hours, 3, 7, 11 and 15 days after the end of the bortezomib cycle.

**Results:**

Autoreactive LLPCs are established in the spleen and bone marrow of SLE-prone mice very early in ontogeny, before week 4 and before the onset of symptoms. The generation of LLPCs then continues throughout life. LLPC counts in the spleen plateau by week 10, but continue to increase in the bone marrow and inflamed kidney. When LLPCs are depleted by the proteasome inhibitor bortezomib, their numbers regenerate within two weeks. Persistent depletion of LLPCs was achieved only by combining a cycle of bortezomib with maintenance therapy, for example cyclophosphamide, depleting the precursors of LLPCs or preventing their differentiation into LLPCs.

**Conclusions:**

In SLE-prone NZB/W F1 mice, autoreactive LLPCs are generated throughout life. Their sustained therapeutic elimination requires both the depletion of LLPCs and the inhibition of their regeneration.

## Introduction

Systemic lupus erythematosus (SLE), the prototype of a systemic autoimmune disease, is characterized by the production of pathogenic autoantibodies that directly or indirectly contribute to the pathogenesis of SLE, resulting in cell destruction and inflammation [1,2]. NZB/W mice spontaneously develop a disease closely resembling human SLE. We have shown before that these mice develop both long-lived and short-lived autoreactive plasma cells, and that long-lived plasma cells (LLPCs) contribute significantly to the production of pathogenic autoantibodies [3]. These LLPCs are able to induce nephritis when transferred into immunodeficient mice [4]. As they are refractory to immunosuppressive drugs (for example, cyclophosphamide, dexamethasone and a combination of the two) and B cell depletion, they represent a therapeutic challenge in the treatment of SLE [3,5,6].

Autoantibodies are detectable years before the clinical onset of SLE in humans [7], and by the age of only 4 weeks in NZB/W mice ([3,8] and unpublished data). Some of these autoantibodies are produced by LLPCs since they do not disappear upon treatment of humans or mice with drugs like cyclophosphamide [3,9] or rituximab [10,11]. However, it remains controversial when this population of refractory LLPCs is established in the course of the disease. We have previously shown that a population of autoreactive LLPCs exists in the spleen and bone marrow by week 24 of life [3]. Whether such population is established early in disease pathogenesis and no longer formed later, when constant generation of short-lived plasma cells (SLPCs) may become a hallmark of pathology [12], remain unclear. Alternatively, it has been proposed that a constant generation and turnover of the LLPC pool may be sustained by B cell hyperreactivity [13,14], but also this hypothesis remains to be elucidated. This is valuable information in order not to miss an ‘LLPC window of opportunity’ at the beginning of the disease. Moreover, although interesting studies showed that B cells are able to repopulate the plasma cell-deficient bone marrow [15], it remains rather unclear whether in autoimmunity LLPCs may be replenished from autoreactive memory B cells after therapeutic depletion of these cells.

Here, we show that LLPC generation starts very early in NZB/W F1 mice, long before clinical onset of disease. Then, LLPC counts in the spleen plateau after about 10 weeks, but those in the bone marrow and inflamed kidney increase over lifetime. When PCs are eliminated by bortezomib [16], LLPC counts recover within 15 days in both the spleen and bone marrow. Thus, for persistent elimination of autoreactive LLPCs, existing LLPCs must be depleted (for example, by a cycle of bortezomib), and their regeneration must be prevented by maintenance therapy. Maintenance therapy could be directed at eliminating precursor cells or preventing their activation. Here, we used a combination of bortezomib with cyclophosphamide as a model to demonstrate that, in contrast to bortezomib or cyclophosphamide alone, this combination therapy achieves sustained elimination of LLPCs.

## Methods

### Mice

Female NZB/W F1 mice were bred and maintained under specific pathogen-free (SPF) conditions at the mouse facility of German Rheumatism Research Centre, Berlin. All experiments were performed according to German guidelines for the performance of animal experiments and approved by the according authority (Landesamt für Gesundheit und Soziales, Berlin).

### Flow cytometry and ELISPOT

Flow cytometric analysis of spleen and bone marrow cells was performed after two weeks of bromodeoxyuridine (BrdU) feeding (see below). Staining was performed as described previously [3]. The following antibodies were used for the analysis: anti-CD138 (2-218, BD Pharmingen, San Diego, CA, USA), anti-MHCII (clone M5/114, DRFZ, Berlin, Germany) and anti-kappa light chain (clone 187.1, BD Pharmingen). Cells were acquired using a FACS BD LSR II or FACSCanto II or LSRFortessa flow cytometer (Becton Dickinson, Franklin Lakes, NJ, USA) and analyzed using FlowJo software (TreeStar, San Carlos, CA, USA). Debris and red blood cells (RBCs) were excluded by electronic gating. As the ratio of kappa- to lambda-producing plasma cells is 20:1 in mice, we used single intracellular staining with anti-kappa light chain to determine the number of plasma cells. Plasma cells were identified as CD138+ and intracellular anti-kappa light chain-positive cells. The staining was initially validated using fluorescence-minus-one (FMO) controls for the two markers. This population presents a clear bimodal expression of the markers, with no overlap of positive and negative populations, not requiring any further control for determination of positive and negative populations [17]. Within the plasma cells population, plasmablasts and newly generated plasma cells were identified as cells with the higher expression of MHC-II. Conversely MHC-II^low^ plasma cells were regarded as LLPCs. The expression of MHC-II on B220-positive cells was used to determine the cutoff between high (same MHC-II expression of B220+ cells) and low (lower MHC-II expression as compared to B220+ cells) MHC-II-expressing plasma cells. BrdU staining was performed with anti-BrdU antibody using the BD Pharmingen BrdU FlowKit™ according to the manufacturer’s recommendations.

Enzyme-linked immunospot (ELISPOT) analysis to determine the absolute numbers of anti-double-stranded DNA (anti-dsDNA)-specific antibody-secreting cells (ASCs) was performed as described previously [3,18]. Briefly, ELISPOT plates (MultiScreen™ HTS Filter Plates; Merck Millipore, Darmstadt, Germany) were pre-coated with methyl-BSA (10 μg/ml, Sigma-Aldrich, St. Louis, MO, USA) for 2 h at 37°C and subsequently coated with calf thymus DNA (10 μg/ml; Sigma-Aldrich) overnight. Spleen, bone marrow and kidney single-cell suspensions were filtered twice through a Falcon cell strainer (70 μm), washed and resuspended in RPMI 1640 medium supplemented with 10% fetal calf serum (FCS). After plate blocking, two different cell dilutions were pipetted onto the plates and incubated for 3 h at 37°C in a humid atmosphere with 5% CO_2_. After extensive washing, plates were incubated with biotin-labeled goat anti-mouse immunoglobulin G (IgG) and immunoglobulin M (IgM) (1 μg/ml, Southern Biotech, Birmingham, AL, USA) for 1 h and followed by ExtrAvidin™-alkaline phosphatase (Sigma-Aldrich) for 20 min. The spots were developed with NBT/BCIP (Thermo Fisher Scientific, Waltham, MA, USA) and enumerated using an automated ELISPOT reader and software (AID Diagnostika, Strassberg, Germany).

### Cell count

Absolute cell numbers of spleen, bone marrow and kidneys were assessed by automatic cell counting either by Schaerfe CASY Cell Counter (Schaerfe System GmbH, Reutlingen, Germany) or by MACSQuant Analyzer (Miltenyi Biotec, Cologne, Germany). Absolute plasma cell numbers were calculated based on total cell numbers (by automatic cell counting) and frequencies of cell types in each organ measured by flow cytometry. For ELISPOT assay, the absolute numbers of ASCs per organ were calculated by the number of spots/well multiplied by the dilution factor of the cell suspension used for the assay. The total number of bone marrow cells was calculated based on the assumption that both femoral bones contain approximately 8% of total bone marrow cells [16].

### LLPC generation

#### BrdU pulse-chase experiments

In the experiments analysing the generation of LLPC over the lifetime mice of different ages (4 to 32 weeks) were given BrdU in their drinking water (protected from light, 1 mg/ml + 1% glucose) for two weeks in order to determine the time point of formation of the LLPC compartment. This made it possible to discriminate between cells generated during and before the feeding period. As shown previously, this two-week feeding interval is sufficient for discriminating between short- and long-lived plasma cells [3].

In the experiments dissecting the capacity to generate LLPC at different age, mice were continuously fed BrdU for six weeks from the ages of 4 to 10 weeks (young) and 20 to 26 weeks (old). Six weeks after the end of the feeding period, the number of surviving BrdU-positive plasma cells was measured. These cells are LLPCs that were generated during the feeding period and survived the next six weeks.

#### Autoreactive LLPCs formation

To characterize the establishment kinetics of autoreactive (anti-dsDNA) LLPCs, plasmablasts and SLPCs were depleted in NZB/W F1 mice at different ages (4, 6, 8 and 20, 24, 30 weeks of age) by administering four injections of cyclophosphamide (35 mg/kg bw) within two weeks [3]. After treatment, the remaining anti-dsDNA-specific LLPCs were enumerated in spleen and bone marrow by ELISPOT.

### Plasma cell depletion

Twelve- to 16-week-old NZB/W F1 mice with an established plasma cell pool received a single bortezomib cycle consisting of two intravenous (i.v.) injections (0.75 mg/kg bw) of bortezomib over a 36-hour interval. For the combination treatment, mice were additionally treated with 35 mg/kg bw cyclophosphamide alone or in combination with the starting of the bortezomib cycle. Cyclophosphamide was re-administered every four days until the end of the experiment. Twelve hours and 3, 7 and 15 days after the last bortezomib injection, the mice were sacrificed and their spleen and bone marrow cells were characterized and quantified by flow cytometry and ELISPOT. MHC-II expression on the plasma cells population was analyzed to discriminate between plasmablasts and newly generated plasma cells (MHC-II^high^) and mature LLPCs (MHC-II^low^) in these experiments [3]. As previously shown, expression of MHC-II correlates well with the expansion phase of the plasma cell population and can be used as a surrogate marker to distinguish proliferating plasmablasts from noncycling plasma cells [3,19]. This is in accordance with the observation that all BrdU-negative LLPCs expressed little MHC-II and BrdU-positive SLPCs expressed high levels of this molecule, with the limitation that in the BrdU-positive population of SLPCs also some MHC-II ^low^ cells are included, which may be newly generated LLPCs.

### Statistical analysis

Statistical analysis was performed using GraphPad Prism (GraphPad Software Inc., San Diego, CA, USA). If not otherwise indicated, the indicated numbers are means with standard error of the mean (SEM). Statistical significance was calculated using the unpaired two-tailed Student *t* test. *P* values <0.05 were regarded as significant.

## Results

### LLPC generation in SLE-prone mice

#### The generation of LLPCs in NZB/W F1 mice starts early in ontogeny and continues through life

To analyze the generation of LLPCs in SLE-prone NZB/W F1 mice over their lifetime, young (age: 4, 6, 8, and 12 weeks) and old (age: 24, 26 and 27 weeks) mice received BrdU (1 mg/ml) in their drinking water for two weeks. BrdU-negative CD138+ plasma cells (those which had not performed DNA synthesis during the two weeks of BrdU feeding) were classified as long-lived [3].

**Figure 1:**
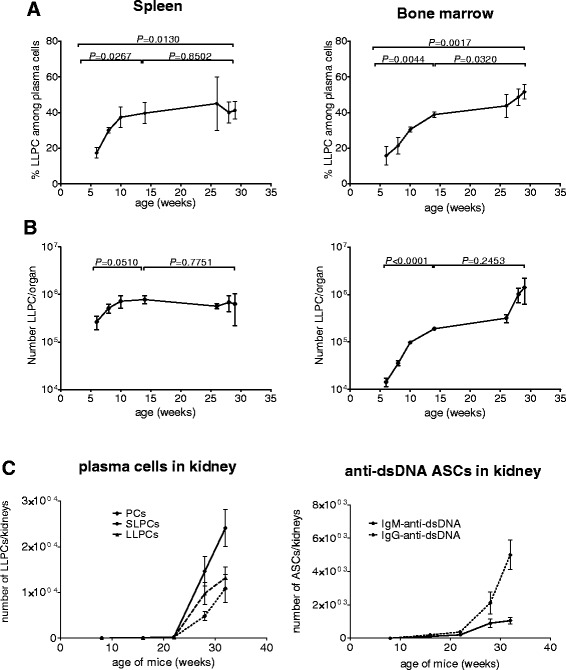
**The majority of long-lived plasma cells (LLPCs) are generated by the age of 14 weeks in NZB/W mice. (A)** Age-dependent frequencies of bromodeoxyuridine (BrdU)-negative LLPCs of total CD138+ plasma cells and **(B)** absolute numbers of BrdU-negative LLPC per organ. Mice of 4, 6, 8, 12 and 24, 26, 27 weeks of age were fed BrdU continuously for two weeks and then subjected to BrdU incorporation analysis of splenic and bone marrow plasma cells. n = 4 mice per time point. **(C)** Absolute numbers of total plasma cells (PCs), BrdU-negative LLPC and BrdU-positive short-lived plasma cells (SLPC) measured by flow cytometry (left) and double-stranded DNA (dsDNA)-specific antibody-secreting cells (ASCs) measured by enzyme-linked immunospot (ELISPOT) (right) in the kidneys of NZB/W mice at different ages. Mice of 6, 14, 20, 26 and 30 weeks of age were fed BrdU continuously for two weeks and then subjected to BrdU incorporation analysis, and anti-dsDNA-specific ELISPOT of kidney plasma cells. n = 4-11 mice per time point. Data are presented as mean and standard error of the mean (SEM ). Numbers and horizontal bars represent *P* values from statistical comparison between time points by two-tailed unpaired *t* test.

In six-week-old mice, 17% (±3%) of all plasma cells in the spleen were LLPCs, which were initially generated before the age of 4 weeks (Figure 1A), long before the clinical onset of disease. The frequency of LLPCs increased until 10 weeks of age, then reached a plateau and remained without significant changes until the end of the observation period (29 weeks of age). At that time, all of the mice had full-blown disease. In terms of absolute cell counts, the numbers of splenic LLPCs increased by 250,000 cells on average between weeks 6 to 8, by another 207,000 by week 10, and by a further 60,000 by week 14 (Figure 1B). Then the number of LLPCs in the spleen did not change significantly between week 14 and week 29. In the bone marrow, the LLPC frequency increased from 16% (±5%) at 6 weeks of age to 38% (±1%) of all plasma cells at 14 weeks (Figure 1A). From then, until 29 weeks of age, LLPC frequencies increased to 45 to 50% of all plasma cells. The absolute numbers of bone marrow LLPCs increased on average by 22,000 cells between weeks 6 to 8, by another 62,000 cells between weeks 8 to 10, and by 92,000 between weeks 10 to 14 (Figure 1B). Thereafter, the numbers of LLPCs increased further by on average 128,000 between weeks 14 and 24 and by 1,082,000 between weeks 26 to 29. Therefore, LLPC generation starts very early in ontogeny, that is, before the onset of symptoms.

Plasma cells may also accumulate in the kidney of NZB/W F1 mice with full-blown disease [14,18,20]. To analyze the kinetic of accumulation of LLPCs in the kidney, mice of 6, 14, 20, 26 and 30 weeks of age were fed BrdU continuously for two weeks and then subjected to BrdU incorporation analysis and the absolute numbers of total plasma cells, BrdU-negative LLPCs and BrdU-positive SLPCs were measured by flow cytometry. Plasma cells appear in the kidney from week 16 of age, and then accumulate without reaching a plateau (Figure 1C). The number of LLPCs increased on average from 51 cells at 16 weeks of age to 13,181 cells at 32 weeks of age in the two kidneys. The number of SLPCs increased on average from 56 cells at 16 weeks of age to 10,900 cells at 32 weeks of age. Accordingly, the number of autoreactive plasma cells secreting antibodies against ds-DNA (IgG and IgM isotypes) measured by ELISPOT, constantly increased in the kidney from week 16, peaking at the end of the observation period (32 weeks of age) without reaching a plateau.

Therefore, the generation of LLPCs continues throughout life and, at later stage of disease, LLPCs persistently accumulate in the bone marrow and kidney but not in the spleen.

#### The generation of LLPCs continues with age and disease progression

**Figure 2:**
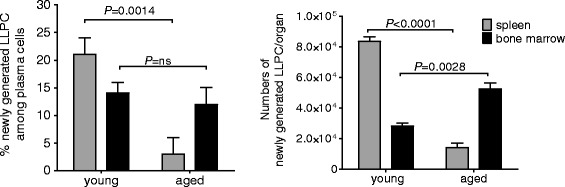
**Generation of long-lived plasma cells (LLPCs) continues with age and disease progression.** Frequencies (left) and absolute number (right) of bromodeoxyuridine (BrdU)-positive newly generated LLPCs enumerated in the spleen and bone marrow of young and old mice. Young (4 weeks of age) and old (20 weeks of age) NZB/W F1 mice were fed BrdU for six weeks, and BrdU incorporation studies were performed six weeks after the end of BrdU feeding. Cells were gated on CD138 + BrdU+ lymphocytes, which are newly generated LLPCs formed during the BrdU feeding period. Data are presented as mean and standard error of the mean (SEM). n = 5 mice. Numbers and horizontal bars represent *P* values from statistical comparison between groups by two-tailed unpaired *t* test. n.s., nonsignificant.

In order to understand whether the capacity to form LLPCs changes with age and disease phase, four-week-old NZB/W F1 mice (without signs of disease activity and very low levels of dsDNA-antibodies) and 20-week-old NZB/W F1 mice (with mild disease, low proteinuria, and high autoantibody titers) were fed BrdU for six weeks (from weeks 4 to 10 or from weeks 20 to 26 of age, respectively). BrdU feeding was then stopped and animals were kept for another six weeks without further BrdU feeding. The spleen and bone marrow of the mice were then analyzed for persistent, BrdU-labeled cells. These BrdU-positive cells, which must have been generated during the preceding BrdU feeding period, can thus be regarded as LLPCs that were generated in the feeding period. Twenty-one ± 3% (83,487 ± 3,018 cells/organ) and 14 ± 2% (28,300 ± 1,821 cells/organ) of the plasma cells in spleen and bone marrow of 16-week-old mice, respectively, were LLPCs that had been generated between weeks 4 and 10 of age (Figure 2). In 32-week-old mice, 4.3 ± 0.9% (14,302 ± 2,756 cells/organ) of splenic plasma cells and 13 ± 1.7% (52,362 ± 4,028 cells/organ) of bone marrow plasma cells were LLPCs generated between weeks 20 to 26 of age (Figure 2). Thus, LLPCs accumulate in the spleen mostly early in life (until week 10), but accumulate in the bone marrow throughout life, at constant rates.

#### Generation of autoreactive LLPCs

**Figure 3:**
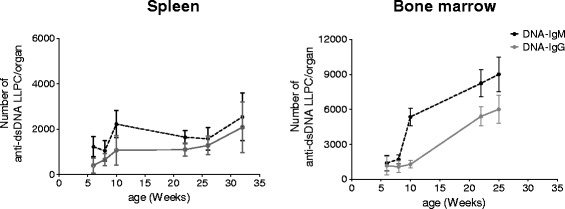
**Autoreactive long-lived plasma cells (LLPCs) are early and continuously generated in NZB/W mice.** Absolute numbers of cyclophosphamide-resistant LLPCs secreting double-stranded DNA (dsDNA)-specific antibody in spleen and bone marrow. NZB/W F1 mice at 4, 6, 8 and 20, 24, 30 weeks of age were treated with four injections of cyclophosphamide (35 mg/kg bw) within two weeks in order to deplete plasmablasts and short-lived plasma cells [3]. After treatment, the remaining anti-dsDNA-specific LLPCs were enumerated by enzyme-linked immunospot (ELISPOT). Data are presented as mean and standard error of the mean (SEM). n = 4-5 mice per time point.

To understand whether the generation of autoreactive LLPCs followed the same kinetics as compared to total LLPCs, SLPCs were eliminated from the analysis by injecting four doses of cyclophosphamide in the two weeks prior to the analysis [3]; the remaining dsDNA-specific LLPCs in the spleen and bone marrow were enumerated by ELISPOT. In the spleen, IgM dsDNA-specific LLPCs increased 1.8-fold between weeks 6 to 10 of age and then reached a plateau (0.7 fold change) between weeks 10 to 32 (Figure 3). Splenic IgG dsDNA-specific LLPCs increased 2.6-fold between weeks 6 to 10 of age, plateaued at week 22, and later increased 2-fold between weeks 22 to 31 (Figure 3). In the bone marrow, however, IgM dsDNA-specific LLPCs increased 3.8-fold between weeks 6 to 10, and further increased at constant rates until week 26. IgG dsDNA-specific LLPCs in the bone marrow showed this same steep increase, but delayed. Between 6 to 10 weeks of age, the numbers remained stable, but increased 4-fold at constant rates from 10 to 26 weeks of age (Figure 3).

This demonstrates that the generation of autoreactive LLPCs starts early in life and that autoreactive LLPCs continue to accumulate at constant rates in the bone marrow of NZB/W mice throughout life.

### Depletion and regeneration of LLPCs

**Figure 4:**
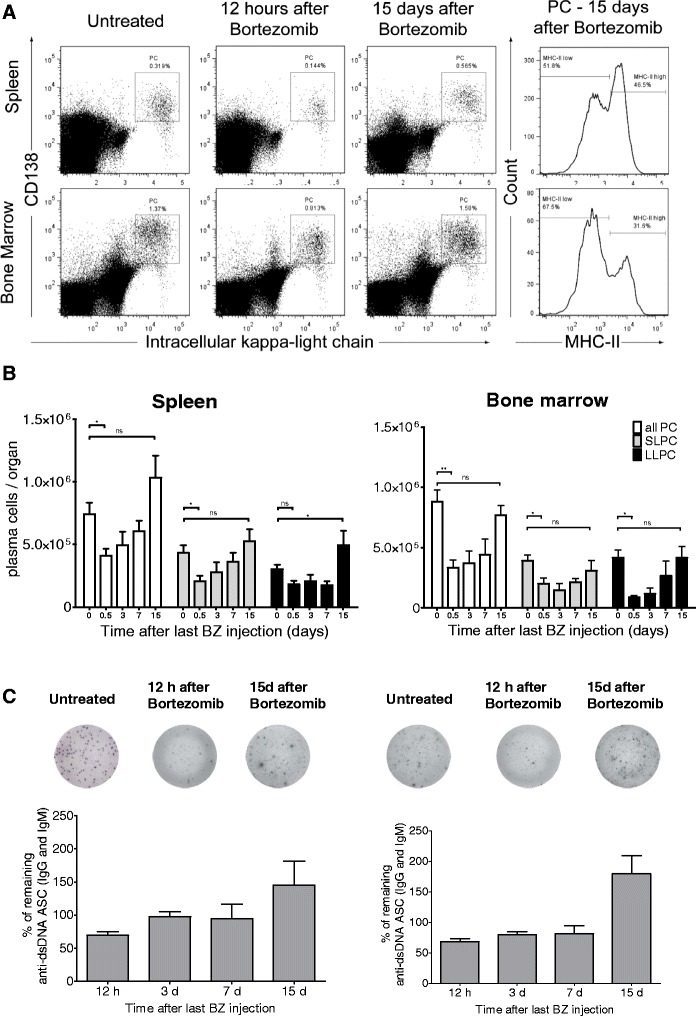
**Bortezomib-mediated long-lived plasma cell (LLPC) depletion is transient.** Twelve- to 16-wk-old NZB/W F1 mice were treated with one bortezomib cycle consisting of two bortezomib injections within 36 h and analyzed 12 h and 3, 7 and 15 days after the last bortezomib injection. **(A)** Representative gating strategy for plasma cell analysis by flow cytometry. Lymphocytes were gated according to their position in forward and sideward scatter, and doublets were excluded from the analysis (not shown). Plasma cells were identified as CD138+ and intracellular kappa light chain + cells both in spleen and bone marrow. Within the plasma cells population, plasmablasts and newly generated plasma cells were identified as cells with a higher expression of MHC-II. Conversely, MHC-II^low^ plasma cells were regarded as LLPCs. A representative plot of the expression of MHC-II on the plasma cell population is shown for spleen and bone marrow 15 days after bortezomib treatment. **(B)** Absolute numbers of all CD138+/intracellular kappa light chain + plasma cells (white bars) and the subgroups of short-lived, MHC^high^ (gray bars) and long-lived, MHC-II^low^ (black bars) plasma cells in spleens (left) and bone marrows (right) 12 h (0.5 days), 3, 7 and 15 days after one cycle of bortezomib measured by flow cytometry. n = 7 mice per time point. **(C)** Frequencies of the remaining anti-double-stranded DNA (dsDNA) antibody-secreting cells (ASCs) (immunoglobulin G (IgG) and immunoglobulin M (IgM) combined) in spleen (left) and bone marrow (right) calculated by comparison with untreated mice (day 0), as detected by enzyme-linked immunospot (ELISPOT). n = 4 mice per time point. Data are presented as mean and standard error of the mean (SEM). n.s., nonsignificant. *P* >0.05, ^*^ *P* ≤0.05, ^**^ *P* ≤0.01 by two-tailed unpaired *t* test.

LLPCs of NZB/W F1 mice were depleted by a single bortezomib cycle consisting of two injections of bortezomib (0.75 mg/kg bw; 36-hour interval) [16]. Twelve hours after the last injection, plasma cell counts had decreased on average from 741,949 to 410,617 in the spleen (72 ± 42.4% decrease) (*P* = 0.0161) and from 881,886 to 332,620 in the bone marrow (74.1 ± 30% decrease) (*P* = 0.0014) (Figure 4A and B), but recovered later. On day 15 after bortezomib treatment, splenic and bone marrow counts had reached 140% and 70% of the number in untreated mice (day 0), respectively (Figure 4B). On average about 50% and 68% of newly generated plasma cells (day 15) in the spleen and bone marrow, respectively, were LLPCs according to expression of MHC class II (Figure 4A). Twelve hours after the last bortezomib injection, autoreactive dsDNA-specific plasma cells (enumerated here as ASCs either secreting IgG- or IgM-dsDNA antibodies) in spleen and bone marrow were depleted up to 50% of the number in untreated mice. However, 15 days later, they regenerated, reaching a higher number as compared to untreated mice in both the spleen and bone marrow (Figure 4C). Interestingly, the depletion of anti-dsDNA plasma cells was not as efficient as the depletion of total plasma cells. The ELISPOT assay in this experiment was designed for the enumeration of all the ASCs producing either IgM- or IgG-dsDNA antibodies. Further experiments showed that ASCs producing IgM-dsDNA are particularly resistant to bortezomib-mediated depletion (with only 40 to 50% reduction) whereas IgG-dsDNA ASCs are efficiently depleted (70 to 80% reduction) (manuscript in preparation).

### Persistent depletion of autoreactive LLPCs

**Figure 5:**
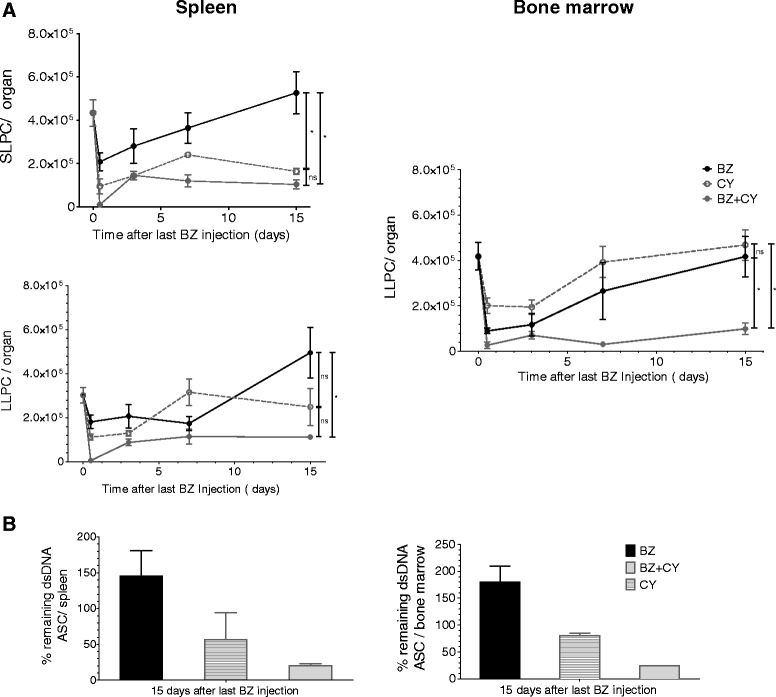
**Persistent depletion of long-lived plasma cells (LLPCs) using cyclophosphamide in combination with bortezomib.** Twelve- to 16-wk-old NZB/W F1 mice were treated with: (1) one bortezomib cycle (black dots and black line); (2) intraperitoneal injection of 35 mg/kg bw cyclophosphamide on day 0, 3, 7 and (open dots, dotted line); (3) one bortezomib cycle combined with cyclophosphamide on day 0, 3, 7 and 11 (gray dots, connecting line). **(A)** Absolute numbers of short-lived MHC^high^ in the spleen and long-lived MHC-II^low^ plasma cells in the spleen (left) and bone marrow (right) 12 h and 3, 7, and 15 days after the last bortezomib injection measured by flow cytometry. **(B)** Frequencies of the remaining anti-double-stranded DNA (anti-dsDNA)-secreting cells in the spleen (left) and bone marrow (right) calculated by comparison with untreated mice (day 0), as detected by enzyme-linked immunospot (ELISPOT) 15 days after the last bortezomib injection. n = 4 mice treated with bortezomib, n = 3 with cyclophosphamide and n = 4 with bortezomib plus cyclophosphamide. Data represent mean and standard error of the mean (SEM). Numbers and horizontal bars represent *P* values from statistical comparison between groups at the end of the observation period (15 days after the bortezomib cycle). n.s., nonsignificant. *P* >0.05, ^*^ *P* ≤0.05, ^**^ *P* ≤0.01 by two-tailed unpaired *t* test.

Twelve- to 16-week-old NZB/W F1 mice were treated with a cycle of bortezomib, cyclophosphamide alone or with a combination of the two. Cyclophosphamide was injected intraperitoneally (i .p.) on day 0, 3, 7 and 11 after the bortezomib cycle in order to deplete plasma cells and, on the other hand, to block their regeneration (Figure 5). We have previously shown that cylophosphamide can eliminate SLPCs from the spleen but that LLPCs in spleen and bone marrow (including anti-dsDNA ASCs) survive the treatment [3,5]. Plasma cell numbers in the spleen and bone marrow were determined 12 hours and 3, 7 and 15 days after the last bortezomib treatment. Confirming our previous results [3,5], the treatment with four doses of cyclophosphamide alone induced a significant reduction of SLPC numbers and only a slight decrease of splenic LLPC numbers without affecting LLPCs in bone marrow (Figure 5A). The combination of a cycle of bortezomib with cyclophosphamide induced a further tendential reduction of splenic LLPC numbers at the end of the observation period (from an average of 248,667 in cyclophosphamide-treated mice to 111,708 in mice treated with the combination therapy). Importantly, at the end of the observation period (15 days after bortezomib treatment) in the bone marrow, the combination of bortezomib with cyclophosphamide maintained the LLPC numbers significantly lower as compared to the groups treated with the single therapy (on average 98,844 LLPCs in the bone marrow of mice treated with the combination therapy as compared to 468,667 and 417,637 LLPCs in mice treated with single cyclophosphamide or bortezomib therapy, respectively) (Figure 5A). Accordingly, 15 days after the last bortezomib injection, plasma cells secreting autoantibodies binding to dsDNA (enumerated here as ASCs either secreting IgG or IgM-dsDNA antibodies) were depleted to 19 ± 6% in spleen by bortezomib and cyclophosphamide and only to 56 ± 37% in mice treated only with cyclophosphamide (Figure 5B). In bone marrow, anti-dsDNA plasma cells were depleted to 24 ± 0.5% and only to 80 ± 5% in mice treated only with cyclophosphamide (Figure 5B). Thus, combination treatment consisting of a single cycle of bortezomib to deplete plasma cells together with continued suppression of LLPC regeneration by cyclophosphamide results in persistent ablation of autoreactive plasma cells.

## Discussion

Here, we showed for the first time that the generation of autoreactive LLPCs in SLE-prone NZB/W mice starts very early in life, long before the onset of disease. Already mice at the age of 4 weeks had anti-dsDNA-secreting plasma cells resistant to cyclophosphamide in both bone marrow and spleen. In the bone marrow, autoreactive LLPC counts increased at a stable rate over life without reaching a plateau, even in six-month-old mice. In the spleen, LLPC numbers increased only in the first 12 weeks and then remained stable over time. This demonstrates that autoreactive LLPCs are constantly generated in NZB/W mice and that, later in life, they accumulate in the bone marrow and inflamed kidney but not in the spleen. These findings pose a therapeutic challenge since LLPCs are resistant to conventional immunosuppression (for example, high-dose cyclophosphamide and/or dexamethasone) or B cell-depletion strategies [3,5,6]. They can be efficiently ablated by proteasome inhibition with bortezomib [16]. However, after depletion of LLPCs with bortezomib, the LLPC counts recovered within 15 days reaching the levels of untreated mice. Thus, for maintained ablation of autoreactive plasma cells, the regeneration of LLPCs must be blocked as well. Here, we showed that depletion of plasma cells by bortezomib in combination with a maintenance therapy to prevent the regeneration of autoreactive LLPCs results in persistent ablation of autoreactive LLPC in NZB/W mice.

Are these findings of any relevance for human autoimmune diseases?

SLE autoantibodies are considered to be pathogenic in human SLE [1], and some of them can be produced by LLPCs especially in refractory patients, as determined based on their resistance to cyclophosphamide and B cell-depleting therapy as with rituximab [6,9]. Moreover, their titers increase over time in active disease [1], indicating continued generation of autoreactive LLPCs. Remarkably, plasma cell generation has been identified as a marker of active disease [21-23]. Furthermore, elimination of all plasma cells including LLPC and B cells (that is, their precursors) by anti-thymocyte globulin (ATG) followed by autologous stem cells transplantation leads to long-term remission in SLE patients [9]. All of this evidence strongly suggests that the continuous generation of autoreactive LLPCs, their important role in disease pathogenesis and the need of targeting B cells and plasma cells for the therapeutic elimination of the autoreactive LLPCs, are modeled by NZB/W mice.

Notably, the dynamics of generation and maintenance of autoreactive LLPCs is not only determined by the rate of generation, but also by the capacity of the body to support LLPCs in the long run. It has been shown that the number of plasma cells in human and mouse bone marrow is determined by the number of chemokine (C-X-C motif) ligand 12 (CXCL12)-expressing stromal cells, which organize survival niches for individual LLPCs [24]. The frequency of such stromal cells in the bone marrow is approximately 1%; accordingly, the physiological frequency of bone marrow plasma cells is also about 1% [25]. Thus, about 10^9^ and 10^6^ LLPCs can be hosted in the bone marrow of healthy humans or healthy mice, respectively [12,25]. Here we found that the maximum capacity to support LLPC survival in the spleen is reached after only 12 weeks, in line with our previous findings [3]. In the bone marrow, this number was reached after 29 weeks (the end of the observation period). Importantly, LLPCs accumulate in the inflamed kidney of SLE mice at a later stage of the disease, confirming that nephritic kidneys can provide survival niches for LLPCs [14,18,20], increasing the capacity to support LLPC survival during disease. In humans, the situation may be slightly different in that a patient’s capacity to host LLPCs in the bone marrow may be reached before disease onset or early in disease. Nevertheless, the new LLPCs can be generated and maintained efficiently, likely by newly formed plasmablasts outcompeting LLPC for their survival niches [12,26] or, most probably, by homing of new autoreactive LLPCs in new niches in inflamed tissues as shown by us and others for SLE mice [14,18,20].

Our findings have relevant clinical implications. As discussed above, the existence of autoreactive LLPCs is a therapeutic challenge. Considering the very early accumulation of autoreactive LLPCs in the bone marrow and spleen of NZB/W mice, our data suggest that a ‘clinically relevant window of opportunity’ for preventing the accumulation of autoreactive LLPCs would exist only for the kidney. However, persistent activation and accumulation of autoreactive LLPCs in the bone marrow may cause relapses in patient with SLE even after long periods of clinical inactivity [6,27]. Therefore other therapies aimed at targeting LLPCs are needed. LLPCs can be eliminated efficiently by ATG [9], anti-lymphocyte function-associated antigen 1 (LFA1) plus anti-very late antigen-4 (VLA4) [15], transmembrane activator and calcium modulator and cyclophilin ligand interactor-immunoglobulin (TACI-Ig) [28] and bortezomib [16] also in advanced stage of the disease. However, therapeutic ablation via these approaches has two big disadvantages. First, it is not selective for autoreactive LLPCs, but also eliminates protective LLPCs in all cases [9]. Second, in lasting immune reactions and in autoimmune reactions, LLPC counts quickly recover within four weeks [15] or two weeks, as shown here for NZB/W mice and suggested recently for SLE patients [29]. Moreover, continued elimination of plasma cells cannot be a preferred therapeutic option since that would imply long-term immunodeficiency with the complete absence of humoral immunity and increased infection-related mortality [30]. Here, we suggest an alternative approach combining plasma cell ablation therapy with follow-up treatment to suppress the regeneration of autoreactive LLPCs. In the case of bortezomib, this also would have the additional benefit of reducing unwanted side effects like neurotoxicity and thrombocytopenia [31]. It could be argued that also this combination therapy with agents targeting plasma cells and B cells may promote an indiscriminate ablation of auotoreactive as well as protective antibodies with the obvious caveats regarding a higher risk of infection. Notably, we described that bortezomib treatment of patients with SLE induced a greater reduction in pathogenic antibody titers (anti-dsDNA antibodies 58.7% reduction) than protective ones (for example anti-tetanus antibodies 29.2% reduction) ([29] and Alexander *et al.*). Moreover, the administration of cyclophosphamide for immunosuppression, as used here, is only a proof of principle. Other more fitting options are available for patients (that is, depleting B cells with anti-CD20 or targeting B cell differentiation into plasma cells and survival with anti-BAFF). In particular, combined LLPCs targeting and anti-BAFF therapy (that is, using the approved drug belimumab) might be a first efficient way to eliminate LLPCs and on the other hand to interfere with their regeneration and persistence, with the advantage to contrast the increased level of BAFF described after B cell depletion [32,33]. Notably, the use of belimumab instead of a complete B cell-depletion therapy (for example, using rituximab) could help to preserve protective memory B lymphocytes promoting the regeneration only of the protective LLPC compartment. Indeed, treatment with belimumab is associated with significant reductions in the numbers of transitional, naive and activated B cells, as well as CD20 + CD138+ plasma cell precursors (plasmablasts) [34-36]. Conversely, the number of memory B cells and T cell is preserved after belimumab therapy indicating that these cells are independent of BAFF for survival [37]. Consistent with the preservation of memory B cells and T cells, belimumab treatment does not substantially affect preexisting anti-pneumococcal or anti-tetanus toxoid antibody levels with similar rates of serious and/or severe infections as compared with the placebo-treated group [34,38-40]. These results, together with the described greater reduction of autoreactive antibodies after bortezomib in SLE, let us speculate that belimumab combined with bortezomib treatment might not compromise the immune response to infection dramatically and indiscriminately. Finally, the combination of LLPC ablation with immunosuppression would also open options for the selective recovery of protective humoral immunity, for example, by vaccination or transfer of autologous, protective plasma cell precursors. Therefore, our results strongly suggest that when transferring such plasma cell depletion strategies to humans, combining plasma cell ablation with an efficient, preferably selective, ablation of the precursors of autoreactive LLPCs could be useful.

## Conclusions

This study explored the generation and maintenance of autoreactive LLPCs in SLE-prone NZB/W mice and the kinetic of regeneration of these cells after depletion. We showed that autoreactive LLPC generation starts very early in life, long before the onset of disease and that these cells are constantly generated during disease. Later in life, LLPCs accumulate in the bone marrow and inflamed kidney but not in the spleen. When LLPCs are depleted by the proteasome inhibitor bortezomib, their numbers regenerate within two weeks, due to constant B cell hyperactivity. Persistent depletion of LLPCs was achieved only by combining a cycle of bortezomib with maintenance therapy, for example cyclophosphamide, depleting the precursors of LLPCs or preventing their differentiation into LLPCs. Our results provide clear evidences that targeting LLPCs in isolation would have limited efficacy in autoimmune disease. This study enters into the recent debate concerning which B cell subset should be targeted in SLE [13,27,32,41], and strongly suggests that combining plasma cell ablation with an efficient, preferably selective, ablation of the precursors of autoreactive LLPCs could represent a new useful strategy in antibody-mediated autoimmune diseases.

## Abbreviations

Anti-dsDNA: anti-double-stranded DNA
ASCs: antibody-secreting cells
ATG: anti-thymocyte globulin
BrdU: bromodeoxyuridine
CXCL12: chemokine (C-X-C motif) ligand 12
IgG: immunoglobulin G
IgM: immunoglobulin M
LFA1: lymphocyte function-associated antigen 1
LLPC: long-lived plasma cell
SLE: systemic lupus erythematosus
SLPC: short-lived plasma cell
TACI-Ig: transmembrane activator and calcium modulator and cyclophilin ligand interactor-immunoglobulin
VLA4: very late antigen-4
